# Point-of-Care Lateral Flow Assays for Tuberculosis and Cryptococcal Antigenuria Predict Death in HIV Infected Adults in Uganda

**DOI:** 10.1371/journal.pone.0101459

**Published:** 2014-07-07

**Authors:** Yukari C. Manabe, Bareng A. S. Nonyane, Lydia Nakiyingi, Olive Mbabazi, Gloria Lubega, Maunank Shah, Lawrence H. Moulton, Moses Joloba, Jerrold Ellner, Susan E. Dorman

**Affiliations:** 1 Infectious Diseases Institute, College of Health Sciences, Makerere University, Kampala, Uganda; 2 Division of Infectious Diseases, Department of Medicine, Johns Hopkins University School of Medicine, Baltimore, Maryland, United States of America; 3 Department of International Health, Johns Hopkins Bloomberg School of Public Health, Baltimore, Maryland, United States of America; 4 Department of Microbiology, College of Health Sciences, Makerere University, Kampala, Uganda; 5 Boston University School of Medicine and Boston Medical Center, Boston, Massachusetts, United States of America; National HIV and Retrovirology Laboratories, Canada

## Abstract

**Background:**

Mortality in hospitalized, febrile patients in Sub-Saharan Africa is high due to HIV-infected, severely immunosuppressed patients with opportunistic co-infection, particularly disseminated tuberculosis (TB) and cryptococcal disease. We sought to determine if a positive lateral flow assay (LFA) result for urine lipoarabinomannan (LAM) and cryptococcal antigenuria was associated with mortality.

**Methods:**

351 hospitalized, HIV-positive adults with symptoms consistent with TB and who were able to provide both urine and sputum specimens were prospectively enrolled at Mulago National Referral Hospital in Uganda as part of a prospective accuracy evaluation of the lateral flow Determine TB LAM test. Stored frozen urine was retrospectively tested for cryptococcal antigen (CRAG) using the LFA. We fitted a multinomial logistic regression model to analyze factors associated with death within 2 months after initial presentation.

**Results:**

The median CD4 of the participants was 57 (IQR: 14–179) cells/µl and 41% (145) were microbiologically confirmed TB cases. LAM LFA was positive in 38% (134), 7% (25) were CRAG positive, and 43% (151) were positive for either test in urine. Overall, 21% (75) died within the first 2 months, and a total of 32% (114) were confirmed dead by 6 months. At 2 months, 30% of LAM or CRAG positive patients were confirmed dead compared to 15.0% of those who were negative. In an adjusted model, LAM or CRAG positive results were associated with an increased risk of death (RRR 2.29, 95% CI: 1.29, 4.05; *P* = 0.005).

**Conclusions:**

In hospitalized HIV-infected patients, LAM or CRAG LFA positivity was associated with subsequent death within 2 months. Further studies are warranted to examine the impact of POC diagnostic ‘test and treat’ approach on patient-centered outcomes.

## Background

In sub-Saharan Africa (SSA), tuberculosis (TB) is responsible for considerable morbidity and mortality [1]. Hospitalized patients in SSA continue to suffer high mortality (range 30–45%); the majority of these patients are HIV-infected and die from undiagnosed and untreated opportunistic infections [2]–[6]. Autopsy studies have shown that many of these severely immunosuppressed patients die of potentially treatable infections, particularly disseminated TB and cryptococcal infection, but the infectious diagnosis at autopsy was not clinically considered during hospitalization [7]. Co-infection with more than one opportunistic pathogen may also contribute to mortality [8]. Among hospitalized HIV-infected adults with fever and/or sepsis syndrome, significant rates of TB have been noted (often disseminated) as well as invasive fungal disease [9], [10].

Several studies have examined predictors of mortality in hospitalized HIV patients or among TB suspects and have found a significant proportion of patients have disseminated tuberculosis with mycobacteremia (range 13–42%) which may present with only fever progressing to septic shock [3], [9], [11]–[14]. Mycobacteremia doubled the risk of in-hospital death from 22% to 44% (OR1.97, 95% CI = 1.19, 3.27, *P* = 0.026) in a study of patients who fulfilled systemic inflammatory response syndrome (SIRS) criteria for sepsis [9]. Unfortunately, mycobacterial blood culture is slow as a diagnostic due to the 20 hour doubling time of *Mycobacterium tuberculosis* (Mtb). Mortality has also been associated with enzyme-linked immunoassay detection of urinary LAM, a glycolipid component of the Mtb cell wall that is excreted in urine [2], [15]; this suggests that urinary LAM may be detected in patients with disseminated disease and may be a good surrogate marker for mycobacteremia as well as death.

In the pre-antiretroviral therapy era, cryptococcal disease was one of the leading contributors to death in HIV-infected adults [16] and remains a significant opportunistic infection in SSA [17], [18]. Cryptococcal antigenemia is an independent predictor of death in patients with low CD4 T cell counts about to initiate ART [19]–[21]. In a survey of bacterial and fungal infections in hospitalized, HIV-infected adults in Tanzania, 11% were found to have *Cryptococcus neoformans* infection [10], and TB and cryptococcal infection were both important causes of hospitalized illness and death.

Rapid point-of-care tests (POC) that fulfill the ASSURED criteria (affordable, sensitive, specific, user-friendly, robust/rapid, equipment free, deliverable to those who need the test/no refrigeration requirement) are very attractive in resource-limited settings [22]. Recently, several new POC lateral flow assays have been developed that have high sensitivity in this population of immunosuppressed HIV-infected patients. The urinary lipoarabinomannan (LAM) lateral flow assay (LFA) (Determine TB LAM Ag, Alere, Waltham, MA, USA) is a point-of-care test that has the highest sensitivity in patients with disseminated TB [23]–[26]. Among patients with CD4 T cell counts less than 100 cells/µl, the sensitivity ranged from (52%–59%), with uniformly high specificity (>94%). In another South African study of mostly hospitalized patients suspected to have extrapulmonary and disseminated TB, the sensitivity in patients with a CD4 T cell count <100 cells/µl was 82.6%, specificity 93% [27]. The cryptococcal antigen LFA (Immuno-Mycologics, Inc. Norman, OK, USA) is specific and easy to use [28], and in urine, the sensitivity was 91% compared to serum. We sought to determine risk factors for early death (within 2 months) in hospitalized HIV-infected adults in Uganda, with a particular focus on understanding whether lateral flow assays for TB and cryptococcal disease diagnosis can identify patients at risk for early death.

## Methods

### Study Participants

506 TB suspects were prospectively enrolled at the Infectious Disease Institute (IDI) HIV clinic or at Mulago National Referral Hospital in Kampala, Uganda between January, 2011 and November 2011 as part of a study to evaluate the accuracy of a lateral flow immunochromatographic test (Determine TB LAM Ag) to detect mycobacterial LAM in urine [29]. All subjects were documented to be HIV-positive, at least 18 years of age, and suspected to have active TB with at least one of the following: cough, fever, night sweats, weight loss. Patients who had taken more than 2 days of TB treatment in the 60 days prior to screening or who were unwilling or unable to provide a urine specimen were excluded. This analysis focused on the subset of 351 hospitalized patients who were able to provide both sputum and urine samples for the main diagnostic accuracy study.

### Procedures

All patients had demographic details recorded and underwent a standardized questionnaire related to the signs and symptoms of TB at enrollment. Two sputum specimens were collected for direct smear stained with the auramine O method and examined by fluorescence microscopy (FM), and then were processed using standardized protocols in the mycobacteriology lab. After sodium hydroxide and N-acetyl-L-cysteine decontamination, centrifuged pellets underwent FM smear, then solid (Lowenstein-Jensen slant) and liquid (Mycobacterial Growth Indicator Tube [MGIT], Becton Dickinson, Sparks, MD) mycobacterial culture. Excess pellet material was stored frozen. Mycobacterial cultures that were positive for acid fast bacilli underwent Capilia TB (Tauns Laboratory, Inc., Kamishima, Izunokuni, Japan) testing for Mtb complex identification. The frozen centrifuged remaining pellet was also analyzed using Xpert MTB/RIF according to the manufacturer's instructions. Whole blood was cultured for mycobacteria in the MYCO/F LYTIC (Becton and Dickinson; blood inoculation volume 3 ml) or BacT/ALERT MB (BioMerieux, Marcy-l'Etoile, France; inoculation volume 5 ml) tubes. CD4 T cell count and chest x-ray were also obtained at enrollment. Urine was collected for urine Determine TB-LAM lateral flow assay. For this assay, 60 µl was pipetted onto the sample pad. According to the manufacturer's instructions, the strip was read 25 minutes later by an independent technician who compared the test strips with the reference card provided by the manufacturer and graded the result from 1+ to 5+. If the positive control bar was not visible, then the strip was invalid and the test repeated. The remainder of the urine was heated to 95°C for 30 minutes using a boiling water bath or dry heating block, centrifuged at 10,000 rpm for 15 minutes, and supernatants pipetted off and frozen. Frozen thawed urine was also batch tested using the FDA-approved cryptococcal antigen (CRAG) LFA (Immy Inc., Norman, Oklahoma, US) according to the manufacturer's instructions. Briefly, 40 µL of urine was placed in an Eppendorf tube. The CRAG LFA dipstick was placed in the tube and allowed to incubate at room temperature for 10 minutes. The qualitative results were interpreted as follows: a single control line was negative, two lines visible were interpreted as positive, and if the control line failed to develop, the test was invalid and was repeated.

Participants had a scheduled two-month follow-up study visit if their initial urinary LAM test was positive and all other mycobacterial testing was negative. All other patients had a review of their medical records and were contacted (by phone call or home visit) at 2 months and then at 6 months to determine their final TB status (dead, alive, unknown, diagnosed and treated for TB).

### TB status definitions

Participants who had Mtb cultured from any specimen were defined as confirmed TB. Possible TB was defined as a patient who had no culture positive, plus one or more of the following criteria: sputum smear microscopy positive but no sputum culture positive for Mtb, empiric TB treatment with documented clinical improvement, diagnosis of active TB per a non-study clinician, death reported to be due to active TB per medical source. Patients were classified as having no evidence of TB if they did not meet the criteria for either of the above.

### Statistical Analysis

Descriptive statistics were used to characterize the hospitalized study population. We fitted a multinomial logistic regression model for a three-category outcome of dead, alive or status unknown at 2 months, with the alive status as the reference category. We first fitted this model to be consistent with a low-resource scenario where no other tests were available on admission (such as CD4 T cell count) except the LAM LFA, the cryptococcal LFA, and direct sputum smear. The multinomial regression model was fitted with the main predictor as a combination of LAM or CRAG result. This provided the relative risk ratio of being a) dead or b) lost to follow-up compared to those who were alive as predicted by the LAM positivity and adjusted for baseline characteristics. In a secondary analysis, we adjusted for CD4 category. We also constructed Kaplan-Meier curves of survival probability versus time-to-death by LAM positivity, with Cox model estimation of hazard ratios. Participants who could not be traced at 2 months were considered lost to follow-up on the day after enrollment.

### Ethics Statement

The study was approved by the institutional review boards in Uganda, Johns Hopkins University, and Boston Medical Center. Written informed consent was obtained from all study subjects prior to enrollment into the study.

## Results

### Patient characteristics

**Figure 1:**
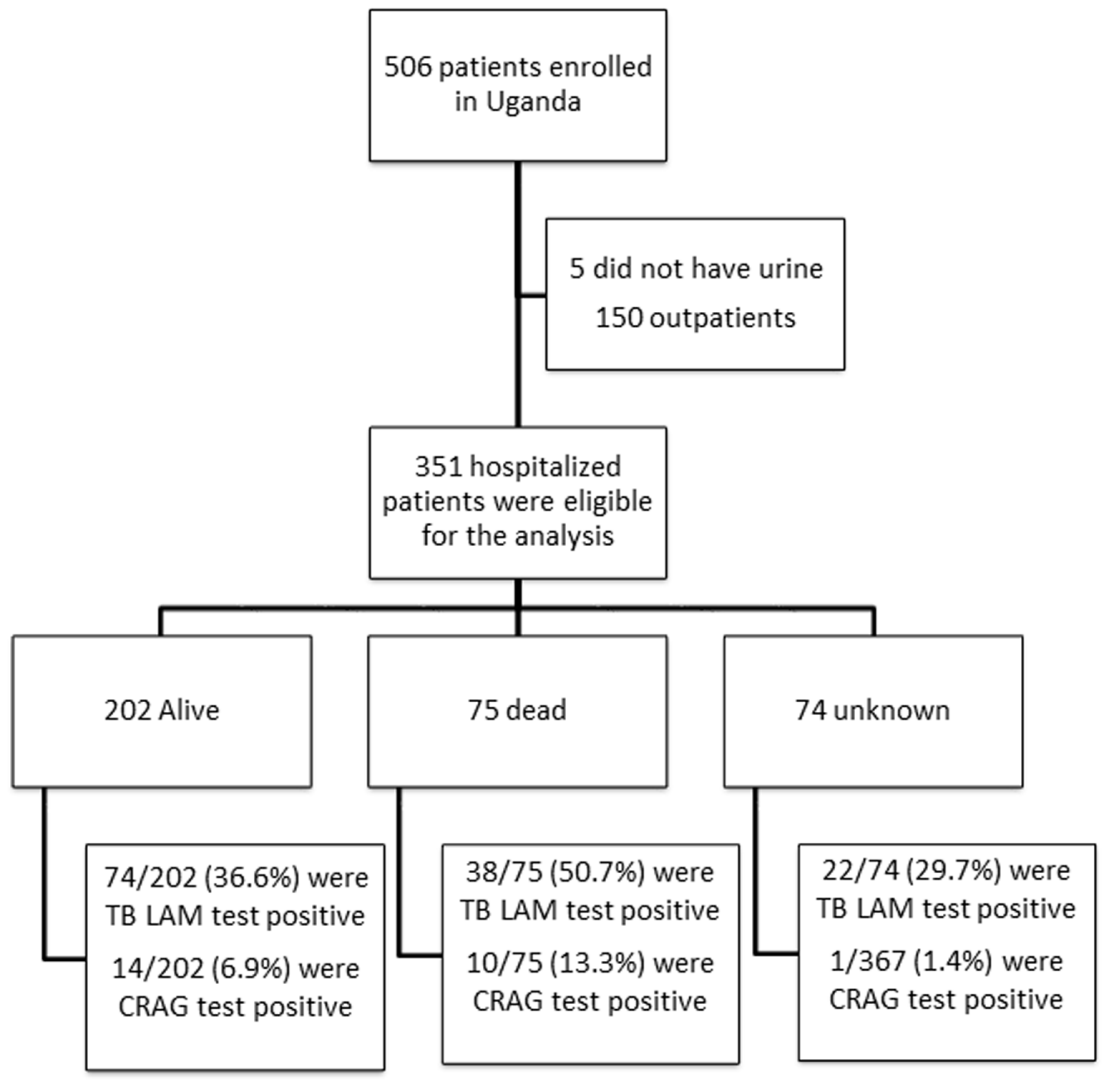
Study participant flow chart.

Table 1 shows the characteristics of the 351 hospitalized participants included in this analysis. (Figure 1) Of these, 64.4% (226) were women and the median age was 31 years (IQR: 27–38). The median CD4 was 57 (IQR: 14–179) cells/µl; 47.6% (167) of participants had a CD4<50 cells/µl and 37.6% (132) of participants were on antiretroviral therapy at enrollment. The majority (96.9%) of patients had cough of any duration; only 62.7% had cough more than 14 days. Fevers (94.1%) and weight loss (96.6%) in the previous 4 weeks were present in the majority of the patients.

**Table 1:** Baseline (enrollment) clinical characteristics and laboratory testing results of the hospitalized study population Uganda (N = 351).

Characteristic	Total N = 351 (% or IQR)	Alive N = 202 (% or IQR)	Dead N = 75 (% or IQR)	Unknown status N = 74 (% or IQR)
Female	226 (64.4)	123(60.9)	53(70.7)	50(67.6)
Median age in years (SD)	31(27,38)	32(7.9)	30(8.8)	31(7.7)
Median Karnofsky score (SD)	60 (5.9))	60(5.6)	60(6.2)	60(6.1))
80–100	113 (32.2	71(35.2)	15(20)	27(36.5)
50–70	238 (67.8)	131(64.9)	60(80)	47(63.5
Median CD4 (IQR) cells/mm^3+^	57 (14,179)	73(18,200)	22(8,72)	90.5(18,195)
CD4 counts (cells/mm^3^) <50	167 (47.6)	89(44.1)	47(62.7)	31(41.9)
CD4 counts (cells/mm^3^) 50–99	42 (12.0)	23(11.4)	11(14.7)	8(10.8)
CD4 counts (cells/mm^3^) 100–199	62 (17.7)	37(18.3)	7(9.3)	18(24.3)
CD4 counts (cells/mm^3^) ≥200	75 (21.4)	50(24.8)	8(10.7)	17(22.9)
On antiretroviral therapy at enrollment	132 (37.6)	81(40.1)	26(34.7)	25(33.8)
On cotrimoxazole prophylaxis at enrollment	299 (85.2)	176(87.1)	67(89.3)	56(75.7)
Cough for more than 14 days	220(62.7)	124(61.4)	49(65.3)	47(63.5)
Fevers-within past 4 weeks	330 (94.1)	189(93.6)	71(94.7)	157(77.7)
Night sweats-within past 4 weeks	274 (78.1)	157(77.7)	56(74.7)	61(82.4)
Weight loss-within past 4 weeks	339 (96.6)	192(95.1)	74(98.6)	73(98.7)
Chest X-ray compatible with TB	207(59.0)	118(58.4)	43(57.3)	46(62.1)
TB treatment ever prescribed	50 (14.3)	37(18.3)	6(8)	7(9.5)
Respiratory specimen smear positive for acid fast bacilli	45 (12.8)	27(13.3)	12(16.0)	6(8.1)
Respiratory specimen culture positive for Mtb*	133 (37.9)	72(35.6)	29(38.7)	32(43.2)
Respiratory specimen culture positive for Mtb but all smears negative	53 (15.1)	30(14.8)	11(14.7)	12(16.2)
Blood culture positive for Mtb**	69 (19.7)	37(18.3)	19(25.3)	13(17.6)
Blood culture positive for non-tuberculous mycobacteria	0	0	0	0
Blood culture positive for Mtb but no respiratory culture positive for Mtb	12 (3.4)	9(4.4)	3(4.0)	0
Any culture positive for Mtb	145 (41.3)	81(40.1)	32(42.7)	32(43.2)
Lateral LAM flow positive ≥2+	90 (25.6)	45(22.2)	28(37.3)	17(23.0)
Lateral LAM flow any band positive	134 (38.2)	74(36.6)	38(50.7)	22(29.7)
Cryptococccal antigen test positive	25 (7.1)	14(6.9)	10(13.3)	1(1.4)
LAM or CRAG antigen test positive	151 (43)	83 (41.1)	45 (60.0)	23 (31.1)
Xpert MTB/RIF test positive	93 (26.5)	54(26.7)	23(30.7)	16(21.6)

Number alive = 202 patients, number dead at 2 months = 75 patients, number unknown status at 2 months = 74 patients, CI = confidence intervals, N = number, IQR = interquartile range, SD = standard deviation, TB = tuberculosis, Mtb = Mycobacterium tuberculosis, LAM = lipoarabinomannan, CRAG = cryptococcal antigen.

*2 results missing.

**7 results missing.

+3 of the patients confirmed alive and 2 of the dead patients at 2 months had CD4 count missing.

### TB and the performance sensitivity of Determine TB LAM for TB disease and urine CRAG in hospitalized patients

Table 1 also shows the Mtb test results of the hospitalized participants. Overall 41.3% (145) were microbiologically confirmed TB cases by either respiratory specimen or blood culture. CXR was available for review for 310 participants; 58.9% of participants had an abnormal CXR consistent with TB. LAM LFA was positive with any band intensity in 134 patients (38.2%) with a sensitivity and specificity for detecting TB given the culture-confirmed TB and “no TB” status of 62.1% (90/145) and 81.1% (150/185) respectively (Table S1 in File S1). Sensitivity and specificity of sputum smear for TB were (31.0% (45 of 145) and 100%); and sputum culture (91.7% and 99.5%). Of the 69 patients with MYCO/F LYTIC blood culture positive for Mtb, 61(88.4%) were urinary LAM positive at any band intensity.

Twenty-five (7.0%) patients were CRAG positive in urine. 151(43%) were positive for either test of which 8 (2%) were positive for both tests.

### Mortality

Table 1 also shows the vital status of the 351 participants in this analysis; 75 (21.4%) died within the first 2 months (9 weeks), 202 (57.6%) were known to be alive at 2 months and 74 (21.1%) were not contactable (unknown status). The baseline characteristics of the non-contactable group were similar to those of the ‘alive’ group. At 6 months, 114 (32%) participants were confirmed to have died.

**Figure 2:**
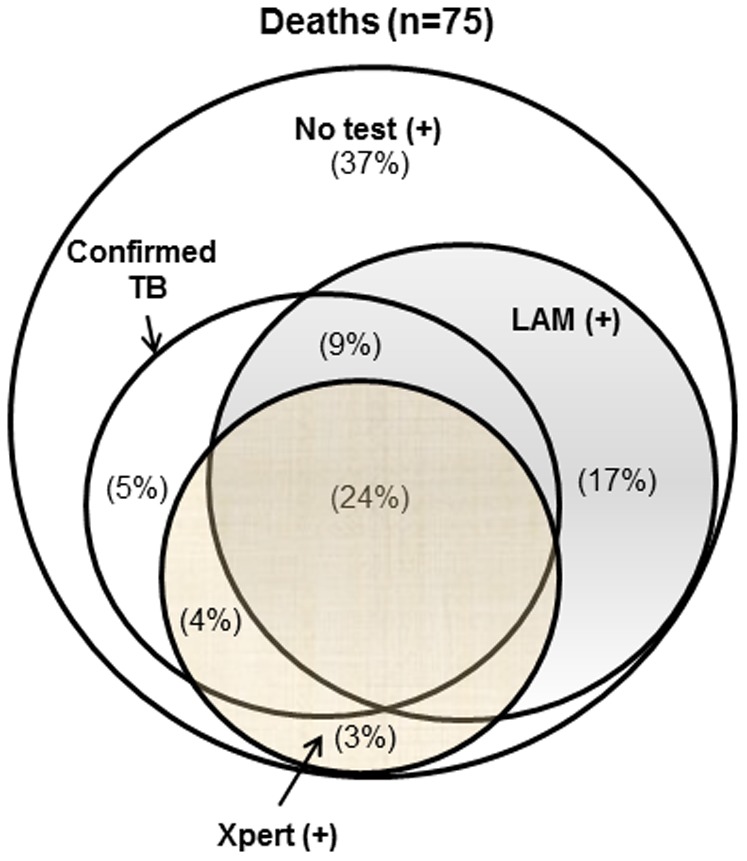
Venn diagram showing the proportions of patients confirmed dead at 2 months (n = 75) who had positive sputum and/or blood cultures (“confirmed TB”), Xpert MTB/RIF positive, and Determine TB LAM LFA positive. Mutually exclusive proportions of patients are shown in each compartment. 17% of the patients who died were LAM positive, but were neither Xpert nor culture confirmed. 24% of the patients who died were positive by all assays.

Of the LAM positive patients, 28.4% (38/134) died by 2 months (22 unknown status), compared to 17.1% (37/217) of the LAM negative patients (52 unknown status). (Table S1 in File S1). Among those with culture-confirmed TB from any site, 28% of those that were LAM-positive died, compared to only 13% of those that were LAM-negative (p = 0.035). Among those in whom no TB diagnosis was made, 34% of those that were LAM-positive died compared to 19% of those that were LAM-negative (p = 0.053). (Table S1 in File S1) Many of the LAM-positive patients with confirmed TB who died were also Xpert MTB/RIF positive (18 of 25). (Figure 2) None of the 12 LAM-positive patients who did not have microbiologically confirmed TB and died were diagnosed by Xpert MTB/RIF. Among all patients at six months, 40% of those that were LAM positive were confirmed to have died, compared to only 28% of the LAM negative patients (p = 0.049). Of the patients with confirmed TB, 39% of those that were LAM-positive died by 6 months compared to 20% of the LAM negative (p = 0.016). (Table S2 in File S1).

Mortality proportions overall and stratified by TB status are presented in the supplemental Tables S1 (2 month mortality) and S2 (6 month mortality). Of the LAM positive patients, 28.4% (38/134) and 40% (54/134) died by 2 and 6 months, respectively, compared to 17.1% (37/217) and 28% (60/217) of the LAM negative patients (P-values<0.05). Among the confirmed TB patients, LAM alone (P-value = 0.035) or LAM or CRAG positive results (P-value<0.001) were associated with an increased risk of death at 2 months. The same results were observed for death at 6 months (LAM positive result, P-value = 0.016 and LAM or CRAG positive results, P-value<0.001).

Many of the urine LAM-positive patients with confirmed TB who died were also sputum Xpert MTB/RIF positive (18 of 25). (Figure 2) None of the 12 LAM-positive patients who did not have microbiologically confirmed TB and died were diagnosed by sputum Xpert MTB/RIF.

**Figure 3:**
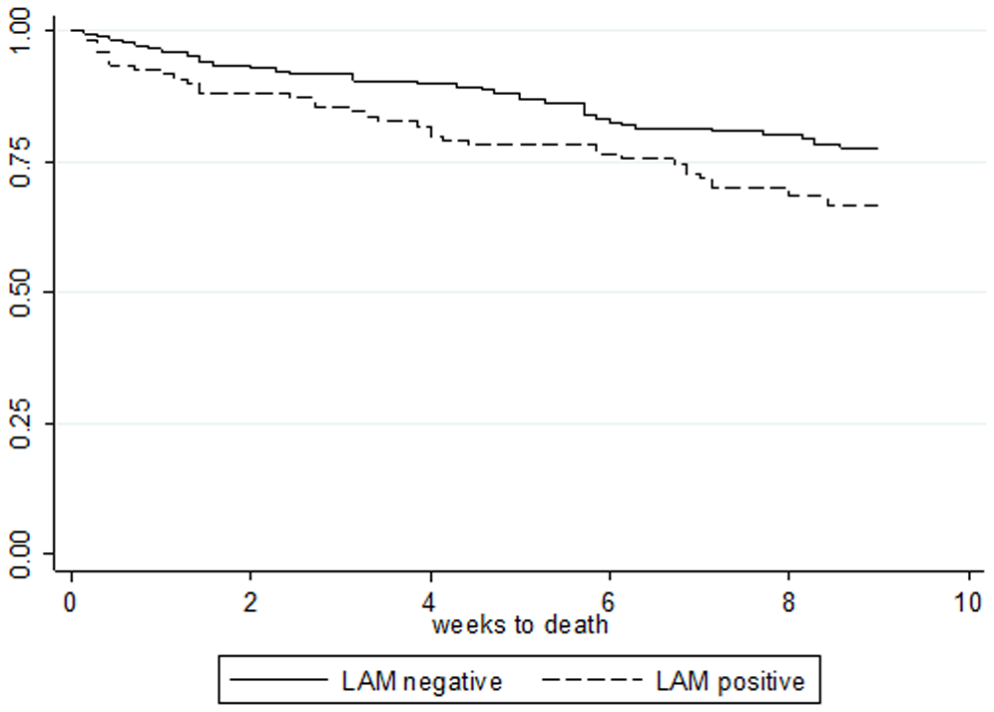
Kaplan-Meier survival curves of LAM negative patients (solid line), and LAM positive patients (dashed line). Participants who could not be traced at 2 months were considered lost to follow-up on the day after enrollment.

The Kaplan-Meier plot by LAM positivity is shown in Figure 3. Patients whose vital status was unknown at 2 months were considered lost to follow-up on day 2. An unadjusted hazard ratio for LAM positivity was 1.67 (*P*-value = 0.025).

### Adjusted multinomial model for factors associated with death within 2 months

The multinomial regression model-adjusted relative risk ratio results for 2-month mortality status are given in Table 2. Relative risk ratios were adjusted for information readily attainable in most hospital settings on admission including gender, age, baseline direct smear positivity and ART status. A LAM or CRAG positive result had a RRR of 2.29(95% CI: 1.29, 4.05; *P* = 0.005). In a secondary analysis adding CD4 T cell count categories to the model, the RRR was attenuated likely due to the interaction between LAM and CD4 results, although there is still a trend toward significance (Table S3 in File S1). Only the lowest CD4 category (<50 cells/µL) remained as an independent, significant predictor of death (RRR 2.71, 95% CI: 1.14, 6.44, *P*-value 0.02), although LAM or CRAG positive results trends towards significant in the multinomial regression analysis (RRR 1.75 (0.94–3.23) P-value = 0.08). (Table 2)

**Table 2:** Adjusted relative risk ratios (RRR), 95% confidence intervals (CI), and P-values for risk factors for death at 2 months*.

Risk Factor	Adjusted RRR of death* (95% CI)	P-value	Adjusted RRR of unknown status* (95% CI)	P-value
Female (reference)	1		1	
Male	0.63(0.35,1.15)	0.13	0.76(0.43,1.35)	0.35
Age<30 years (reference)	1		1	
Age 30–40 years	0.71(0.38,1.15)	0.27	1.18(0.66,2.09)	0.58
Age>40 years	0.89(0.41,1.95)	0.78	0.59(0.24,1.47)	0.26
Sputum smear negative (reference)	1		1	
Sputum smear positive	0.86(0.39,1.90)	0.70	0.64(0.24,1.69)	0.37
No antiretroviral therapy at enrollment (reference)	1		1	
On antiretroviral therapy at enrollment	0.82(0.46,1.45)	0.49	0.74(0.42,1.31)	0.31
No cough more than 14 days (reference)	1		1	
Cough>14 days	1.15(0.6,2.03)	0.63	1.11(0.63,1.94)	0.72
LAM or CRAG antigen test positive	2.29(1.29,4.05)	**0.005**	0.71(0.39,1.28)	0.25

CI = confidence intervals, RRR = relative risk ratio, SD = standard deviation, Mtb = Mycobacterium tuberculosis, LAM = lipoarabinomannan, CRAG = cryptococcal antigen.

*N = 350 with complete records, Multinomial logistic regression model adjusted for gender, age, baseline direct smear positivity, currently on ART. Alive group as reference.

## Discussion

In hospitalized patients, a urinary LAM positive result is associated with death in an adjusted multinomial regression analysis. One-third of the hospitalized, HIV-infected participants in this study died within 6 months of enrollment. These mortality rates are consistent with those previously reported by others [4]–[6], [30] and have not changed appreciably since the beginning of the HIV epidemic in resource-limited settings in SSA. The reasons for this high mortality rate are multifactorial including late HIV presentation, antiretroviral treatment failure, and delays in diagnosis leading to the high acuity of illness in hospitalized patients. Among TB suspects with cough and in a broader category of febrile HIV-positive patients, many studies have documented Mtb infection and mycobacteremia as well as cryptococcal infection as prominent causes of illness and death [3], [9]–[13]. The timely application of new, urine-based lateral flow tests for both TB disease and cryptococcal infection that do not require electricity or technical expertise to perform may avert mortality by accelerating the time to diagnosis.

Among culture-confirmed TB cases who died, LAM testing had additive value to sputum based Xpert testing; 78% were diagnosed by LAM LFA (28% were Xpert negative), and 66% by Xpert (14% were LAM LFA negative). This corroborates the previously published increased sensitivity of sputum smear plus LAM, and Xpert MTB/RIF plus LAM for TB cases overall [24], [31]–[34]. Interestingly, 28% of participants that were LAM-positive died within 2 months compared to 17% of those that were LAM-negative (p = 0.035); all LAM positive cases classified as “no evidence of TB” who died were Xpert negative. Based on our own data and others, urinary LAM is most sensitive in patients with disseminated TB disease [35] who are least likely to be positive by sputum Xpert MTB/RIF and often have atypical symptoms (e.g. fever only) which is often not recognized as TB with concomitant treatment delays [13], [14], [33]. LAM LFA may be more sensitive for an in-patient population with higher mortality risk and may explain why implementation of on-site Xpert MTB/RIF did not impact 2-month mortality in a study done in the same hospital [36].

In our study, 7% of the patients were cryptococcal antigen positive without overt signs of meningitis which is similar to previously reported rates in hospitalized patients in Uganda [30] and not significantly different from the prevalence in ART initiation cohorts (CD4<100 cells/µl) in SSA (Uganda 8.2% [21], South Africa 8.8% [20]). The serum cryptococcal antigen test has high sensitivity and specificity; the urine assay has 91% sensitivity compared to the serum test in published comparative data [28]. Cryptococcal antigenemia is a known independent risk factor for death [19], [20], and pre-emptive treatment with fluconazole can impact outcomes [37]. Testing prior to ART initiation is cost effective using the latex agglutination cryptococcal test and even more cost effective with the new, less expensive lateral flow assay [21], [38].

Our study had several strengths including the prospective collection and testing of urine with LAM LFA in an HIV-infected hospitalized population suspected to have TB in a setting outside of South Africa where many TB diagnostic studies are implemented. Because mortality was not a primary endpoint of the parent study, however, not all patients could be located at 2 months despite rigorous tracing exercises. In addition, we do not have reliable data on which patients received TB treatment which could have altered their outcomes. Secondly, one-third (44/134) of the LAM positive patients were positive at the lowest band intensity. In previous studies, the lowest 1+ band intensity has been difficult to interpret and has had lower specificity than band intensities 2+ and greater [25]. With respect to CRAG detection, the retrospective analysis of previously boiled, freeze-thawed urine could have further decreased the sensitivity of the assay in addition to the use of urine. CRAG titers in urine are considerably lower (22-fold) compared to serum or plasma [28] though this was not clinically significant since the LFA was positive in serum, plasma, and urine in 61 of 62 patients.

Either LAM or CRAG LFA positive results were associated with mortality within 2 months (RRR 2.29, P = 0.005). Our data suggest that the LFA's are diagnosing disease-specific mortality which may be related to disease severity or undiagnosed, untreated disease due to a reference standard that does not capture all patients with true disease (TB sputum culture). Although POC CRAG and LAM may more rapidly diagnose infections that may lead to death in hospitalized patients and have the potential to accelerate timely treatment, the impact on patient centered outcomes needs to be prospectively tested when LAM LFA is used as a screening test in HIV-infected patients and patients are treated promptly. Prospective studies to investigate outcomes with empiric TB treatment as well as the Determine TB LAM test and treat strategies are on-going in sub-Saharan Africa [39]. One observational study from Brazil showed increased mortality with empiric TB treatment, although no weighted adjustment for selection bias was performed in this study [40].

## Conclusions

In hospitalized HIV-positive adults, a positive urinary LAM POC LFA result is associated with mortality. When combined with the CRAG lateral flow POC LFA, the combination (i.e. either test positive) has even higher predictive value. Given the high rates of mortality in hospitalized patients despite the introduction of antiretroviral therapy for HIV, further assessment of the impact of these rapid diagnostics on averting mortality as components of a ‘test and treat’ algorithm is warranted.

## Supporting Information

File S1 **Supporting tables.** **Table S1**, 2 month mortality and LAM/CRAG status stratified by TB categorization. **Table S2**, 6 month mortality and LAM/CRAG status stratified by TB categorization. **Table S3**, Adjusted relative risk ratios (RRR), 95% confidence intervals (CI), and P-values for risk factors for death at 2 months including CD4 category.
(DOCX)
