# Supplementary material for: Point-of-Care Lateral Flow Assays for Tuberculosis and Cryptococcal Antigenuria Predict Death in HIV Infected Adults in Uganda
Source: PLoS One. 2014 Jul 7;9(7):e101459. doi: 10.1371/journal.pone.0101459 (PMC4084886; doi:10.1371/journal.pone.0101459)
Supplement: File S1 — Supporting tables. Table S1, 2 month mortality and LAM/CRAG status stratified by TB categorization. Table S2, 6 month mortality and LAM/CRAG status stratified by TB categorization. Table S3, Adjusted relative risk ratios (RRR), 95% confidence intervals (CI), and P-values for risk factors for death at 2 months including CD4 category. (DOCX) [file pone.0101459.s001.docx]

**Table S1: 2 month mortality and LAM/CRAG status stratified by TB categorization**

|  | Total | | | | Confirmed TB(n=145) | | | | Possible TB (n=21) | | | | No evidence of TB (n=185) | | | |
| --- | --- | --- | --- | --- | --- | --- | --- | --- | --- | --- | --- | --- | --- | --- | --- | --- |
|  | Total | Alive | Unknown | Dead | Total | Alive | Unknown | Dead | Total | Alive | Unknown | Dead | Total | Alive | Unknown | Dead |
|  | 351 | 202(58%) | 74 (21%) | 75 (21%) | 145 | 81(56%) | 32 (22%) | 32(22%) | 21 | 17(81) | 1 (5%) | 3 (14%) | 185 | 104(56%) | 41 (22%) | 40 (22%) |
| LAM positive | 134 | 74(55%) | 22(16%) | **38 (28%)^a^** | 90 | 46(51%) | 19 (21%) | **25 (28%)^a^** | 9 | 7(78%) | 1 (11%) | 1 (11%) | 35 | 21(60%) | **2(6%)^b^** | 12 (34%) |
| LAM negative | 217 | 128(59%) | 52(24%) | **37 (17%)^a^** | 55 | 35(64%) | 13 (24%) | **7 (13%)^a^** | 12 | 10(83%) | 0 (0%) | 2 (17%) | 150 | 83(55%) | **39(26%)^b^** | 28 (19%) |
|  |  |  |  |  |  |  |  |  |  |  |  |  |  |  |  |  |
| LAM or CRAG positive | 151 | 83(55%) | 23(15%) | **45 (30%)^c^** | 94 | 48(51%) | 19 (20%) | **27(29%)^c^** | 9 | 7(78%) | 1(11%) | 1 (11%) | 48 | 28(58.3%) | **3 (6.25%)^d^** | **17 (35%)^d^** |
| Both LAM and CRAG negative | 200 | 119(60%) | 51 (26%) | **30 (15%)^c^** | 51 | 33(65%) | 13 (25%) | **5(10%)^c^** | 12 | 10(83%) | 0 (0%) | 2 (17%) | 137 | 76(56%) | **38(28%)^d^** | **23 (17%)^d^** |

*^a^ P-value ( odds of death in LAM +ve versus LAM –ve, Alive group as reference) =0.035*

*^b^ P-value ( odds of Unknown status in LAM +ve versus LAM –ve, Alive group as reference) =0.024*

*^c^ P-value ( odds of death in LAM_crag +ve versus LAM_Crag –ve, Alive group as reference) <0.01*

*^d^ P-value ( odds of Unknown status in LAM_crag +ve versus LAM_Crag –ve, Alive group as reference) <0.04*

When all patients with symptoms consistent with TB are considered, 28% of the LAM positive patients (38/134) were confirmed to have died by 2 months, compared to 17.1% of the LAM negative patients (37/217). Among those with culture confirmed TB from any site, 28% of those that were LAM-positive died, compared to only 13% of those that were LAM-negative (P=0.035). Interestingly, among those in whom no TB diagnosis was made, 34% of those that were LAM-positive died compared to 19% of those that were LAM-negative. All of the patients with a LAM positive result were Xpert MTB/RIF negative.

**Table S2: 6 month mortality and LAM/CRAG status stratified by TB categorization**

|  | All (n=351) | | | | Confirmed TB (n=145) | | | | Possible TB (n=21) | | | | No evidence of TB (n=185) | | | |
| --- | --- | --- | --- | --- | --- | --- | --- | --- | --- | --- | --- | --- | --- | --- | --- | --- |
|  | Total | Alive | Unknown | Dead | Total | Alive | Unknown | Dead | Total | Alive | Unknown | Dead | Total | Alive | Unknown | Dead |
|  | 351 | 163(46%) | 74(21%) | 114 (32%) | 145 | 97(46%) | 99 (69%) | 46(31%) | 21 | 16(76%) | 1 (5%) | 4(19%) | 185 | 80(43%) | 41(22 %) | 64(35%) |
| LAM positive | 134 | 58(43%) | 22(16%) | **54(40%)^a^** | 90 | 36(40%) | 19(21%) | **35(39%)^b^** | 9 | 6(67%) | 1(11%) | 2(22%) | 35 | 16(46%) | 2 (6%) | 17(49%) |
| LAM negative | 217 | 105(48%) | 52 (24%) | **60(28%)^a^** | 55 | 31(56%) | 13(23%) | **11(20%)^b^** | 12 | 10(83%) | 0 (0%) | 2(17%) | 150 | 64(43%) | 39 (26%) | 47(31%) |
|  |  |  |  |  |  |  |  |  |  |  |  |  |  |  |  |  |
| LAM or CRAG positive | 151 | 63(42%) | 23 (15%) | **65(43%)^c^** | 94 | 38(41%) | 19(20.%) | **37(39%)^d^** | 9 | 6(67%) | 1(11%) | 2(22%) | 48 | 19(40%) | 3 (6%) | **26(54%)^e^** |
| Both LAM and CRAG negative | 200 | 100(50%) | 51 (26%) | **49(25%)^c^** | 51 | 29(57%) | 13(25%) | **9(18%)^d^** | 12 | 10(83%) | 0 (0%) | 2(17%) | 137 | 61(45%) | 38(28%) | **38(28%)^e^** |

^a^ P-value ( odds of death in LAM +ve versus LAM –ve, Alive group as reference) =0.049

^b^ P-value ( odds of Unknown status in LAM +ve versus LAM –ve, Alive group as reference) =0.016

^c^ P-value ( odds of death in LAM_crag +ve versus LAM_Crag –ve, Alive group as reference) =0.003

^d^ P-value ( odds of Unknown status in LAM_crag +ve versus LAM_Crag –ve, Alive group as reference) =0.009

^e^ P-value ( odds of Unknown status in LAM_crag +ve versus LAM_Crag –ve, Alive group as reference) =0.03

Among all patients at six months, 40% of those that were LAM positive were confirmed to have died, compared to only 28% of the LAM negative patients (P=0.049). Among those with culture confirmed TB from any site, 39% of those that were LAM-positive died, compared to only 20% of those that were LAM-negative (P=0.016). Remarkably, nearly 49% of those that were LAM-positive, but had no clinical or microbiological diagnosis of TB, died by 6 months compared to 31% of those without identifiable TB that were LAM-negative. 43% of patients who had a positive LAM or CRAG test died, compared to only 25% of patients who were by negative for both tests (P=0.003).

**Table S3: Adjusted relative risk ratios (RRR), 95% confidence intervals (CI), and P-values for risk factors for death at 2 months including CD4 category***

| **Risk Factor** | **Adjusted**  **RRR of death* (95% CI)** | **P-value** | **Adjusted**  **RRR of unknown status* (95% CI)** | **P-value** |
| --- | --- | --- | --- | --- |
| Female (reference) | 1 |  | 1 |  |
| Male | 0.65(0.36,1.2) | 0.17 | 0.74(0.43,1.33) | 0.32 |
| Age<30 years (reference) | 1 |  | 1 |  |
| Age 30-40 years | 0.69(0.37,1.30) | 0.25 | 1.24(0.69,2.22) | 0.47 |
| Age>40 years | 0.84(0.36,1.96) | 0.69 | 0.66(0.26,1.66) | 0.37 |
| Sputum smear negative (reference) | 1 |  | 1 |  |
| Sputum smear positive | 0.93(0.40,2.13) | 0.86 | 0.67(0.25,1.79) | 0.42 |
| No antiretroviral therapy at enrollment (reference) | 1 |  | 1 |  |
| On antiretroviral therapy at enrollment | 0.88(0.49,1.59) | 0.68 | 0.75(0.42,1.35) | 0.34 |
| No cough more than 14 days (reference) | 1 |  | 1 |  |
| Cough>14 days | 1.07(0.59,1.92) | 0.83 | 1.09(0.62,1.93) | 0.76 |
| CD4 counts (cells/mm^3^) ≥200 | 1 |  | 1 |  |
| CD4 counts (cells/mm^3^) 100-199 | 1.12(0.37,3.43 | 0.84 | 1.47(0.65.3.30) | 0.35 |
| CD4 counts (cells/mm^3^) 51-99 | 2.53(0.97-7.35 | 0.09 | 1.25(0.46,3.40) | 0.67 |
| CD4 counts (cells/mm^3^) <50 | 2.71(1.14,6.44) | **0.02** | 1.20(0.58,2.50) | 0.62 |
| LAM or CRAG antigen test positive | 1.75 (0.94,3.23) | 0.08 | 0.69(0.37,1.30) | 0.25 |

CI=confidence intervals, RRR=relative risk ratio, SD= standard deviation, Mtb=Mycobacterium tuberculosis, LAM= lipoarabinomannan, CRAG= cryptococcal antigen

*N=350 with complete records, Multinomial logistic regression model adjusted for gender, age, baseline direct smear positivity, currently on ART. Alive group as reference.
